# Stab Wound of the Thoracic Duet

**Published:** 1898-09

**Authors:** W. H. Lyne

**Affiliations:** Richmond, Va.; Demonstrator of Surgery and Demonstrator of Normal Histology, Medical College of Virginia: Late Resident Physician City Almshouse Hospital, Richmond; 409 East Grace Street


### TEXAS MEDICAL JOURNAL.

ESTABLISHED JULY, 1885.

PUBLISHED MONTHLY.—SUBSCRIPTION $1.00 A YEAR.

VOL. XIV. AUSTIN, SEPTEMBER, 1898. No. 3.

### Original Contributions.

For the Texas Medical Journal.

### Stab Wound of the Thoracic Duct.—Recovery.

BY W. H. LYNE, M. D., RICHMOND, VA.
Demonstrator of Surgery and Demonstrator of Normal Histology,
Medical College of Virginia; Late Resident Physician
City Almshouse Hospital, Richmond.

Read before the Richmond Academy of Medicine and Surgery,
August 9, 1898.

During my service in the City Almshouse Hospital of this city, many were the unusual, interesting, and instructive cases that came under observation, since this is the only emergency hospital here. Often cases of such rare occurrence befell the lot of the ambulance surgeon as to be regarded as surgical curiosities, chief among which is the following, viz.: a stabwound of the thoracic duct at the base of the neck, the result of a midnight street brawl.

Dr. John A. Wyeth, in his most lucid essays on ligations, describes, on account of the proximity to the cervical blood-vessels, the anatomy of the thoracic duct, which is but little larger than a goose-quill, near its termination, as follows: "On a level with the insertion of the scalenus it arches to the left, crosses in front of the subclavian, in front of the scalenus, behind the internal jugular and curves downward to empty into the subclavian at its junction with the jugular to form the left innominate vein." Posteriorly to the origin of the sterno-mastoid muscle, lies the small anatomic field bisected by the following vital structures—the pneumogastric and phrenic nerves, internal jugular and subclavian veins, subclavian and left common carotid arteries, the thoracic duct and the near-by brachial plexus; a field which the mighty dare but enter after the most careful deliberation and thorough study, yet the would-be assassin's knife plunged amid this net-work of vital structures wounding only the thoracic duct.

On account of the rarity of injury to the thoracic duct, many works on surgery absolutely ignore the subject while others dismiss it with a paragraph.

Very little is recognized in life concerning diseases of the thoracic duct, necropsic findings, however, demonstrating their existence as secondary chiefly to a tubercular condition or a suppuration in some of the near-by viscera or lymphatic glands. Pus, blood, bile, and even calcareous matter and concretions have been found in the duct; a rare case of ossification of the duct has been noted as well as one of gangrene.

Sir Astley Cooper's experiments on animals revealed that gradual compression of the duct resulted in its dilation, whereas rupture resulted if suddenly compressed; during intestinal digestion, a compression of only a few minutes sufficing to effect a rupture, this being readily explained since the duct at this time is normally distended, due to the absorption of the digested fats brought thither by the lymphatics, the sole conductors of this force-producing product. Where pressure is gradual and permanent, a chylous engorgement ensues, resulting in the establishment of a collateral lymphatic circulation. A varicosed thoracic duct, like a varicose vein, is subject to rupture, discharging, according to locality, into or behind the peritoneum, into or behind the pleura, into the posterior mediastinum, or into the bladder; the effusions producing chylous ascites, chylothorax or chyluria, a case of the latter condition existing intermittently for fifty years in a woman.

Several interesting reports of abdominal and thoracic paracenteses have been made, in which the fluid microscopically proved to be chyle, the quantity being enormous. For instance, 289 pints in 22 tappings; in another, 15 gallons in 68 days; and a third in which 11.8 litres were found and withdrawn post mortem from the pleura.

The causes of rupture of the duct are (1) traumatism or (2) obstruction, which is produced, as in other ducts, by *causes*

*from within*, as infiltration or thickening of its walls, stenosis from cicatrical contraction, thrombi, etc., or *causes from without*, as pressure from neoplasms, etc. A cause not common to the obstruction of other ducts, but analagous, is the blocking of the venous outlet, produced not only by a thrombus but by cardiac dilation with its subsequent venous engorgement, which necessarily interferes with the discharge of chyle into the subclavian vein.

A case is reported where a child with a congenital heart lesion subsequently developed an elephantiasic swelling of the right leg with a papular eruption from which exuded a chylous fluid, such eruptions being associated with or alternating in cases of chylous ascites and chyluria. The frequency of concurrent phlebitis and lymphangitis readily explains the old term "milk leg," now known as a result of phlebitis. The association of thoractic duct disease, ascertained post mortem, with other tubercular conditions leads me to attribute the malnutrition and emaciation in this dread malady largely to this non-recognized cause.

Experimental wounds in animals have demonstrated the spontaneous cure of thoractic duct wounds, yet death from inanition is to be expected in the vast majority of cases.

Spontaneous cure is effected by either or both of two ways: (1) by contraction of the unstriped muscular tissue, which is circular but scant near its termination, along with the auxiliary elastic tissue, which is longitudinal; (2) by spontaneous coagulation of chyle, a property acquired after having passed through the mesenteric glands. Not only are the functions and histologic structure of lymphatics and blood-vessels nearly analagous but also the results of wounds of each, longitudinal ones bleeding less freely than transverse, the severed edges being more readily apposed.

As in other ducts, longitudinal wounds in healing are less liable to be followed by stricture. Since the molecular basis of chyle is emulsified fat (this giving it its milky color, being colorless except during intestinal digestion), it becomes patent that a system deprived of this compound as well as its circulating medium, the excess of the albuminous liquor sanguinis, must necessarily suffer; the patient gradually wasting away if the sequela be a stricture or a fistula, or dying from starvation if the duct be completely severed.

The following case is of more than ordinary interest aside from its infrequency, since recovery, followed by no ill effects, resulted:

CASE.—About 1. a. m. May 5, 1896, I was called to an emergency case at one of the police stations. On entering, information was given by some of the officers "that a negro man had been stabbed in the neck and that *white blood, like milk*, was coming from the wound." A thoracic duct injury was suspected by exclusion, but I silently agreed with them that "I had never seen white blood before."

The negro, aged 24, was of splendid physique, being a porter in a large hay and grain establishment.

On examination, an obliqe stab-wound about one inch long, depth unknown, was found above and behind the left clavicle, and parallel with the outer border of the sterno-cleido-mastoid near its attachment; thus from the anatomy of the parts, necessitating a longitudinal wound of the thoracic duct. There had been considerable hemorrhage, which had stopped, and an abundant milky fluid was steadily escaping from the wound. For quite a time I was at a loss as to treatment, but, acting on the advicc once given me by an older physician, "to look wise, say little and do something if necessary," I decided to tampon, which was repeatedly done after having cleansed the wound with a weak, hot, carbolized solution, the packing of iodoform gauze and compress becoming soaked with chyle. On removing the patient to the hospital, the wound was again redressed under scarcely better aseptic surroundings, using a dressing of like character as before. When this dressing was applied, chyle was escaping in good quantity, though the patient had been slowly moved nearly three miles. On removing the dressing during the ward visit about seven hours therafter, the escaping chyle and oozing had completely stopped, and the regulation dressing was reapplied with the approval of the surgeon-in-chief, Dr. J. G. Trevilian.

The patient was allowed a light diet. His recovery was prompt and uneventful, the only untoward symptom being a slight suppuration, the patient being discharged nine days after his admission, complaining only of a slight stiffness of his left arm. The patient was seen August 2, 1898, and was enjoying perfect health, weighing ten pounds more than he ever weighed before.

I regret to state that no specimen of the chyle was secured for microscopic and analytic examination, which would have proved of special interest.*

*Due credit must be allowed Bertrand Dawson, of London, for his exhaustive medical contribution in Vol. IV. of the *Twentieth Century Practice*, Wyeth, Packard, Parks, and the *American Text Book of Surgery*.

409 East Grace Street.

